# Towards a core outcome set for sarcopenia intervention studies: a scoping review identifying the most frequently reported outcomes across randomized controlled trials in sarcopenia

**DOI:** 10.1007/s41999-025-01285-x

**Published:** 2025-08-12

**Authors:** S. Van Heden, Y. M. Chan, Z. Baoubbou, O. Bruyère, J.-Y. Reginster, M. Surquin, D. Sanchez-Rodriguez, C. Beaudart

**Affiliations:** 1 Public Health Aging Research & Epidemiology (PHARE) Group, Research Unit in Clinical Pharmacology and Toxicology (URPC), Department of Biomedical Sciences, Faculty of Medicine, Namur Research Institute for Life Sciences (NARILIS), University of Namur, 5000 Namur, Belgium; 2 Department of Dietetics, Faculty of Medicine and Health Sciences, University Putra Malaysia, 43400 Serdang, Malaysia; 3 Malaysian Research Institute on Ageing, University Putra Malaysia, 43400 Serdang, Malaysia; 4 Department Physical Activity and Rehabilitation Sciences, Research Unit in Public Health, Epidemiology and Health Economics, University of Liège, Liège, Belgium; 5 Protein Research Chair, Biochemistry Department, College of Science, King Saud University, Riyadh, Kingdom of Saudi Arabia; 6 Geriatrics Department, Brugmann University Hospital, Université Libre de Bruxelles, 1020 Brussels, Belgium; 7 Rehabilitation Research Group, Hospital del Mar Research Institute, 08003 Barcelone, Spain

**Keywords:** Sarcopenia, Older people, Core outcome set—outcomes, Interventional, Scoping review

## Abstract

**Objective:**

This study aimed to identify the most frequently reported outcomes in sarcopenia trials, as a basis for developing a Core Outcome Set (COS).

**Material and methods:**

A systematic review was conducted with a comprehensive search of MEDLINE, Embase, and Cochrane Central Register of Controlled Trials from database inception to March 2024 (PRISMA guidelines, PROSPERO: CRD42024525506). Eligible studies were randomized controlled trials (RCTs) on sarcopenia treatment, including patients diagnosed with a consensus definition. All outcomes reported in those studies were extracted and grouped into predefined subcategories.

**Results:**

Among 3985 references, 58 studies (54 unique RCTs) met the inclusion criteria, reporting 214 efficacy and 39 safety outcomes. The most frequently reported outcomes were muscle mass (50 studies, i.e., present in 86.2% of studies), muscle strength (50 studies, 86.2%), physical performance (46 studies, 79.3%), nutrition-related outcomes (29 studies, 50.0%), and fat mass (28 studies, 48.3%). Among the primary outcomes, change in muscle mass was the most commonly reported outcome (23 studies, 39.7%, with appendicular skeletal muscle mass index and fat-free mass as the predominant measures), followed by change in muscle strength (16 studies, 27.6%, mainly assessed by handgrip strength), followed by change in physical performance (13 studies, 22.4%, mainly assessed by gait speed and lower limb physical function). Secondary outcomes included biomarkers, physical status, quality of life, activities of daily living, bone mass, psychological status, cognitive function, and unclassified outcomes.

**Conclusions:**

This review highlights the extensive heterogeneity of reported outcomes in sarcopenia intervention studies and the urgent need for developing a COS.

**Supplementary Information:**

The online version contains supplementary material available at 10.1007/s41999-025-01285-x.

## Key summary points

**Aim:**

As a first step in the development of a Core Outcome Set, we carried out a scoping review using a systematic methodology to identify the most frequently reported outcomes in sarcopenia intervention studies.

**Findings:**

This scoping review identified 253 distinct outcomes, revealing a high degree of heterogeneity across clinical trials. Muscle mass, muscle strength, and physical performance are the most frequently reported outcomes in sarcopenia trials.

**Message:**

A considerable diversity and heterogeneity of reported outcomes were observed in clinical trials, highlighting the urgent need for outcome standardization and the development of a Core Outcome Set specifically for sarcopenia interventions.

**Supplementary Information:**

The online version contains supplementary material available at 10.1007/s41999-025-01285-x.

## Introduction

Sarcopenia, which is defined by the European Working Group on Sarcopenia in Older People (EWGSOP2) as a decline in muscle strength and mass, with physical performance used to assess its severity [1, 2], has been recognized in 2016 as a distinct disease with the allocation of a specific ICD-10-CM [3, 4]. This represented an important step for clinical management and awareness of sarcopenia’s impact on public health. Besides the diagnosis criteria developed by the EWGSOP2, other criteria exist, including those of the Asian Working Group for Sarcopenia (AWGS), the International Working Group on Sarcopenia (IWGS), the Foundation for the National Institutes of Health (FNIH), and the Sarcopenia Definitions and Outcomes Consortium (SDOC) [5]. The lack of a universally accepted definition of sarcopenia complicates its recognition and management worldwide [5]. To address this issue, the Global Leadership Initiative on Sarcopenia (GLIS) was recently established with the aim of harmonizing criteria and defining a universal standard for the disease [5].

Sarcopenia is a prevalent disease affecting 10% (in studies using EWGSOP2 criteria) [6] to 22% (in studies using EWGSOP criteria) [6] of older people aged 65 years and older [6–8]. This prevalence increases with age and is expected to increase significantly by 2050. It has been reported that the number of people presenting from sarcopenia could reach 2.1 billion people worldwide in 2050 [9], making it a major public health problem. Sarcopenia has major consequences for both the individual and the population as a whole, including increases the risk of falls, fractures, hospitalization, functional decline and mortality [10], besides reducing quality of life [11, 12]. It also has a significant economic impact on society [10, 13].

As sarcopenia is a reversible condition [14], interventional research is crucial. Current treatments for sarcopenia focus mainly on exercise, including endurance and resistance training [15, 16], and nutritional strategies, such as supplementation with vitamin D, protein, antioxidants, long-chain polyunsaturated fatty acids, and adequate energy intake [15, 16]. Among these interventions, resistance exercise, along with increased energy and protein intake, are the most widely used and evidence-supported approaches. No pharmacological treatment is currently available, although several molecules, including apelin and irisin, are under investigation [17, 18]. Recently, Doza et al. published a systematic review examining the various Patient-Reported Outcome Measures (PROMs) used in intervention studies focused on managing sarcopenia [19]. The authors identified a diverse range of PROMs, from those assessing health-related quality of life to measures of depressive symptoms, loneliness, and sleep quality. Their findings underscore the presence of heterogeneity in outcome reporting among clinical trials on sarcopenia.

To address this heterogeneity, the development of a Core Outcome Set (COS) is a promising solution. A COS is defined as “an agreed standardized set of outcomes that should be measured and reported, as a minimum, in all clinical trials in a specific disease” [20]. The harmonization of outcomes through a COS has several benefits: (1) Ensure that the selected outcomes are aligned with patient priorities and concerns, making research more patient-centered; (2) Improve transparency and methodological rigor, reducing selective reporting of outcomes and research waste; (3) Facilitate shared decision-making among stakeholders, including trial designers, guideline developers, healthcare providers, scientific societies, funders and regulators. This prioritization helps to allocate resources more effectively to clinically relevant and scientifically robust interventions [20]. Although COS are increasingly used in medical research, they remain relatively new to gerontology and geriatrics, with no COS yet developed for sarcopenia. Given the growth of clinical trials in this field, now is a crucial time to establish one. Developing a core outcome set (COS) in line with best research practices requires a structured stepwise approach. This paper focuses on the crucial first step, identifying and cataloging all outcomes currently measured in sarcopenia intervention trials through a scoping literature review.

### Methods

#### Generality

This scoping review followed the Preferred Reporting Items for Systematic reviews and Meta-Analyses extension for Scoping Reviews (PRISMA-ScR) Checklist [21], ensuring a systematic and transparent approach (Table S1). Additionally, the Core Outcome Set–STAndards for Development (COS-STAD) criteria [22–24] were applied to guide the development and reporting of core outcome sets. To ensure transparency and reproducibility, the study protocol was registered in the PROSPERO database (registration number CRD42024525506). The review also integrated the Core Outcome Measures in Effectiveness Trials (COMET) initiative, the study is available at COMET registration n°2991 (https://www.cometinitiative.org/Studies/Details/2991).

#### Search strategy and study selection

A comprehensive search was conducted, using a detailed search strategy available in appendix (Table S2), in March 2024 in Medline (via Ovid), Embase, and the Cochrane Central Register of Controlled Trials (CENTRAL) to identify all randomized controlled trials (RCTs) evaluating interventions for sarcopenia. Additional sources included manual searches of the bibliographies of relevant articles, a reference search using Web of Science, and consultations with experts in the field. Previous systematic reviews and meta-analyses were also screened for additional references, and clinical trial registries were consulted to identify unpublished studies.

Search results from both electronic and manual sources were imported into Covidence data management software for systematic processing. Covidence is a web-based collaboration software platform that streamlines the production of systematic and other literature reviews.

The study criteria for this scoping review are presented in Table 1.

**Table 1:** Inclusion criteria of the scoping review (population/concept/context, PCC)

Population	Older people aged 60 years of age or over. Studies were included if the mean sample age was above 60 years, or results were reported separately for people aged 60 years or over Sarcopenia consensus definition includes at least a measurement of two key parameters of sarcopenia (e.g., muscle mass + (muscle strength or physical function)). The following definitions were accepted: (1) European Working Group on Sarcopenia in Older People version 1, (2) European Working Group on Sarcopenia in Older People version 2, (3) Foundation for the National Institutes of Health Sarcopenia Project, (4) Asian Working Group on Sarcopenia, (5) Society on Sarcopenia, Cachexia and Wasting Disorders or (6) International Working Group on Sarcopenia, (7) Sarcopenia Definitions and Outcomes Consortium
Concept	Randomized controlled trial, aiming at the management of sarcopenia using any type of intervention (exercises, nutrition, combined exercises & nutrition, pharmacological treatments, and other interventions)
Context	*Inclusion criteria:* Articles published until March 2024 (date when the last bibliographic search is consulted). Only studies written in English were considered eligible for inclusion [25] Only full-text, peer-reviewed publications in indexed journals were included *Exclusion criteria:* Case reports, congress abstracts, reviews, and letters to the editors (unless they contain original data)

**Fig. 1:**
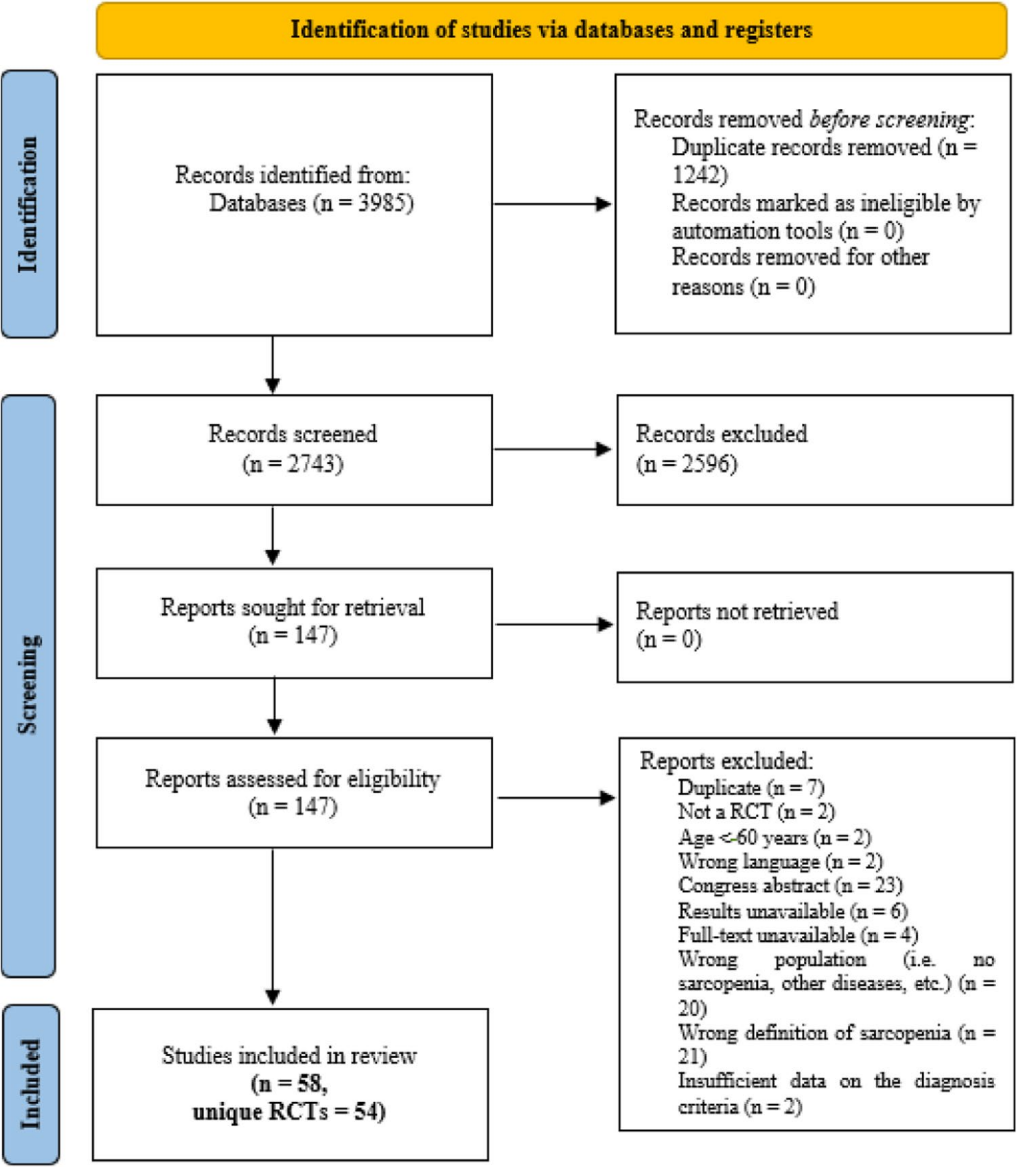
PRISMA 2020 flow diagram

All identified articles were independently screened by reviewers based on title and abstract (CB, DSR, and YMC), followed by a full-text review to assess eligibility. A minimum of two reviewers was required to decide on inclusion or exclusion. In cases of disagreement, a third reviewer was consulted to reach a consensus. The study selection process is summarized in the flowchart (Fig. 1), where exclusion reasons were systematically documented (Table S3).

For a deeper understanding, all relevant documents and supplementary materials have been made available on the Open Science Framework (OSF) platform (https://osf.io/f4zks/).

#### Data extraction & data synthesis

Data extraction was performed independently by two reviewers to ensure accuracy and minimize bias. The extracted data included general study information, such as study characteristics, definitions, and measurement tools for sarcopenia, study design, sample characteristics, study duration, and descriptions of interventions, including exercises, nutrition, combined exercises and nutrition, pharmacological treatments, and comparators. Additionally, primary and secondary outcomes were identified in each study. When this information was not explicitly reported, the study protocols were consulted. The classification of outcomes as primary or secondary was based directly on the terminology used by the original study authors to ensure consistency and respect for the original trial design. An outcome was considered for extraction if it was measured at least twice, before and after the intervention (with the exception of outcomes like adverse events or falls). These outcomes were further categorized into efficacy outcomes, including primary and secondary measures, and safety outcomes. Variables collected for socio-demographic characterization or confounding adjustment were not extracted as outcomes, minimizing the risk of misclassification.

If a RCT was reported in more than one study but with different objectives, each study was treated as a separate entry in the review because the outcomes measured differed depending on the specific aim of each publication.

Each outcome was systematically documented with the following details: the description of the outcome, how it was reported in the clinical trial, its classification based on the 38-outcome list from Dodd et al. (2018) [26], and the measurement tool used. Additionally, supplementary materials were reviewed to identify all available outcomes and ensure comprehensive data extraction. A qualitative synthesis was then performed, summarizing each outcome’s name, outcome’s classification, and measurement tool employed.

To ensure clarity and consistency, the terminology used in clinical trials was standardized during data analysis (e.g., “Jamar hydraulic dynamometer” was categorized as “handgrip test”). Outcomes referring to similar constructions were grouped under broader categories (e.g., “muscle mass” encompassed skeletal muscle mass index, fat-free mass, appendicular skeletal muscle mass, etc.). The terminology used for categorization was selected based on the standardized glossary of terms recommended by the Global Leadership Initiative on Sarcopenia (GLIS) for sarcopenia research [27].

The risk of bias of the included studies was not assessed in this scoping review. Indeed, our aim was not to determine whether a risk of bias might be present in these RCTs or whether it could potentially impact the results. Rather, we simply reported the outcomes measured in the RCTs without interpreting their findings.

#### Summary of results

An analysis was conducted to summarize the frequency of measured outcomes and the distribution of primary outcomes, including their variation according to the type of intervention. Bar charts were used to illustrate these distributions across the included studies.

## Results

After exclusion of duplicates, 2743 studies were screened by title and abstract, and 147 were subsequently assessed in full text to confirm their eligibility. Ultimately, the scoping review included 58 studies drawn from 54 unique RCTs and comprising sub-studies with different objectives derived from the same trials (Fig. 1, flowchart). The reasons for excluding the 89 studies are provided in the supplementary material (Table S3).

### Studies description

Table 2 presents the characteristics of the 54 included RCTs. These RCTs, published between 2012 and 2024, utilized various definitions of sarcopenia, with EWGSOP 2010 being the most frequently applied (20 RCTs, 37.0%), followed by AWGS 2014 (19 RCTs, 35.2%). In terms of sample size, 16 RCTs (29.6%) included fewer than 50 participants, 21 RCTs (38.9%) included between 51 and 100 participants, and 17 RCTs (31.5%) included more than 100 participants. The majority of studies lasted between 10 and 20 weeks (24 RCTs, 44.4%), followed by studies lasting between 20 and 30 weeks (14 RCTs, 25.9%). In terms of interventions, exercise-based interventions were the most common (19 RCTs, 35.2%), followed by a combination of both approaches (17 RCTs, 31.5%), nutritional interventions (11 RCTs, 20.4%), pharmacological interventions (4 RCTs, 7.4%), and other types of interventions (3 RCTs, 5.6%). Geographically, the majority of RCTs were conducted in Asia (31 RCTs, 57.4%), followed by Europe (13 RCTs, 24.1%) and America (7 RCTs, 13.0%). The remaining 3 RCTs (5.6%) were from other regions, including Oceania and various countries. Most of the recruitment was community-based (36 RCTs, 66.7%), and most of the studies were multicenter (33 RCTs, 61.1%). Regarding funding, 22 RCTs (40.7%) received academic funding, and 19 RCTs (35.2%) received industrial funding. The description of each individual RCTs can be found in the supplementary material (Table S4).

**Table 2:** Main characteristics of the 54 included RCTs to treat sarcopenia in older adults

Characteristics	Categories	Number of RCTs (%)
Publication year	< 2015 2015–2019 2020–2024	3 (5.6%) 15 (27.8%) 36 (66.7%)
Definition of sarcopenia	EWGSOP AWGS2014 FNIH EWGSOP2 AWGS2019 Other definition	20 (37.0%) 19 (35.2%) 2 (3.7%) 4 (7.4%) 5 (9.3%) 4 (7.4%)
Number of participants	0–50 51–100 > 101	16 (29.6%) 21 (38.9%) 17 (31.5%)
Duration of the study	< 10 weeks 10 weeks—< 20 weeks 20 weeks—< 30 weeks > 30 weeks	10 (18.5%) 24 (44.4%) 14 (25.9%) 6 (11.1%)
Type of intervention	Exercise Nutrition Exercise + Nutrition Pharmacological treatments Other	19 (35.2%) 11 (20.4%) 17 (31.5%) 4 (7.4%) 3 (5.6%)
Geographical distribution	Asia Europe America Other	31 (57.4%) 13 (24.1%) 7 (13.0%) 3 (5.6%)
Research site	Hospital Community NR	7 (13.0%) 36 (66.7%) 11 (20.4%)
Number of centers	Single center Multiple center NR	14 (25.9%) 33 (61.1%) 7 (13.0%)
Funding	Industry Academic No funding/NR	19 (35.2%) 22 (40.7%) 13 (24.1%)

*AWGS* Asian Working Group for Sarcopenia, *EWGSOP* European Working Group on Sarcopenia in Older People, *FNIH* Foundation for the National Institutes of Health Sarcopenia Project, *NR* not reported, *RCT* randomized controlled trial

#### Outcomes reported in the 58 studies

Given that different outcomes were examined across the individual studies, the outcome analysis was conducted based on the total number of studies identified (n = 58), rather than the number of unique RCTs (n = 54). A total of 253 outcomes were identified across the 58 studies, including 214 efficacy outcomes and 39 safety outcomes. The list and frequency of reporting of the 253 outcomes can be found in the supplementary material (Tables S6, S7 and S8). Among the 214 efficacy outcomes, 13 categories were defined: muscle mass, muscle strength, physical performance, fat mass, bone mass, physical status, biomarkers, psychological status, cognitive function, quality of life, activities of daily living, nutritional outcomes, and other outcomes. On average, 12.1 (± 6.8) outcomes were measured in the articles, with a minimum of 2 outcomes and a maximum of 29 outcomes.

**Fig. 2:**
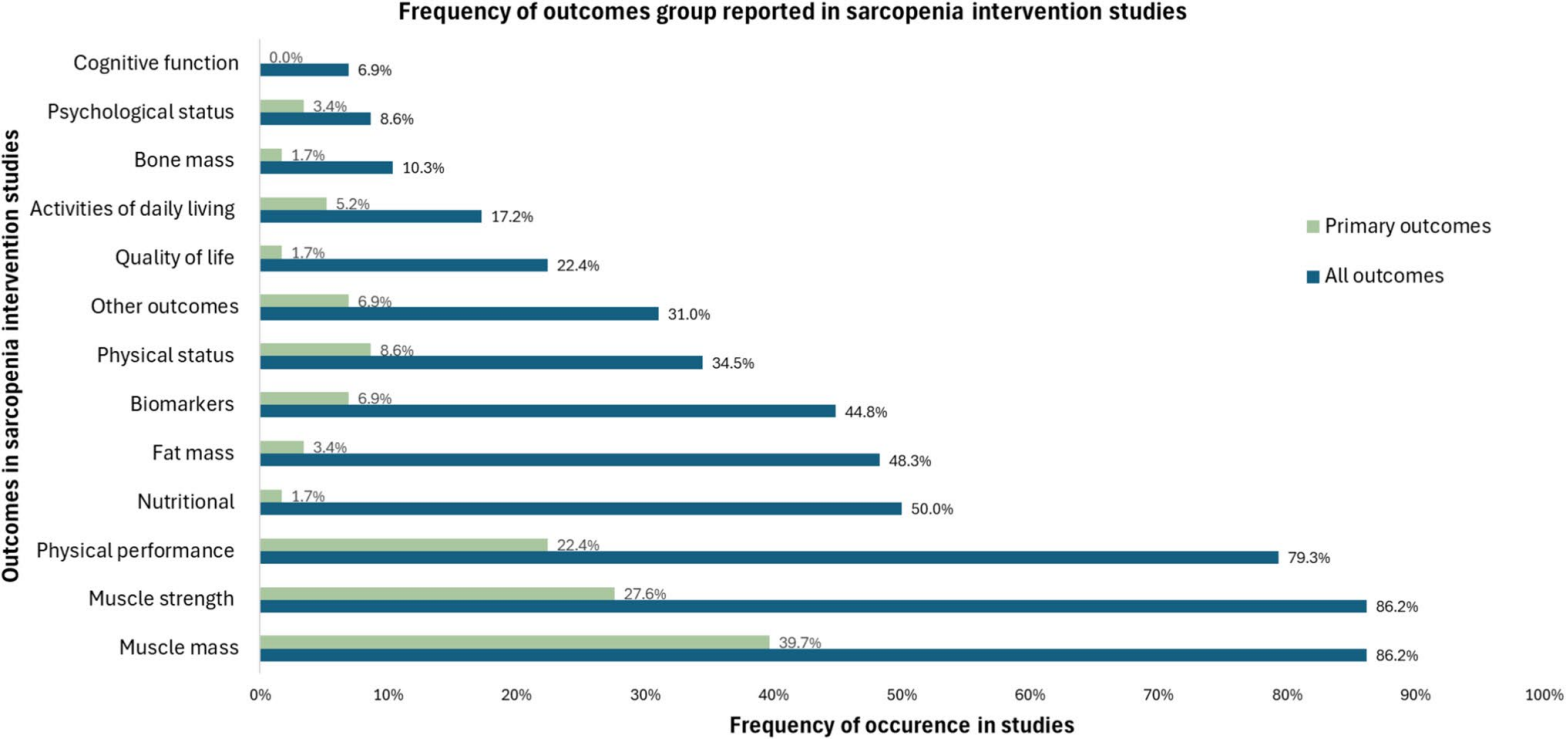
Frequency of reported outcome groups in sarcopenia intervention studies

Change in muscle mass and muscle strength were the most frequently assessed categories, each reported in 50 studies (86.2%). Physical performance was the next most frequently assessed outcome, appearing in 46 studies (79.3%). Nutritional parameters were evaluated in 29 studies (50.0%), followed by fat mass in 28 studies (48.3%), biomarkers in 26 studies (44.8%), and physical status in 20 studies (34.5%). Unclassified outcomes such as falls, sleep, and muscle power were reported in 18 studies (31.0%). Quality of life was assessed in 13 studies (22.4%), and activities of daily living in 10 studies (17.2%). The least frequently assessed categories were bone mass (6 studies, 10.3%), psychological status (5 studies, 8.6%), and cognitive function (4 studies, 6.9%) (Fig. 2).

**Fig. 3:**
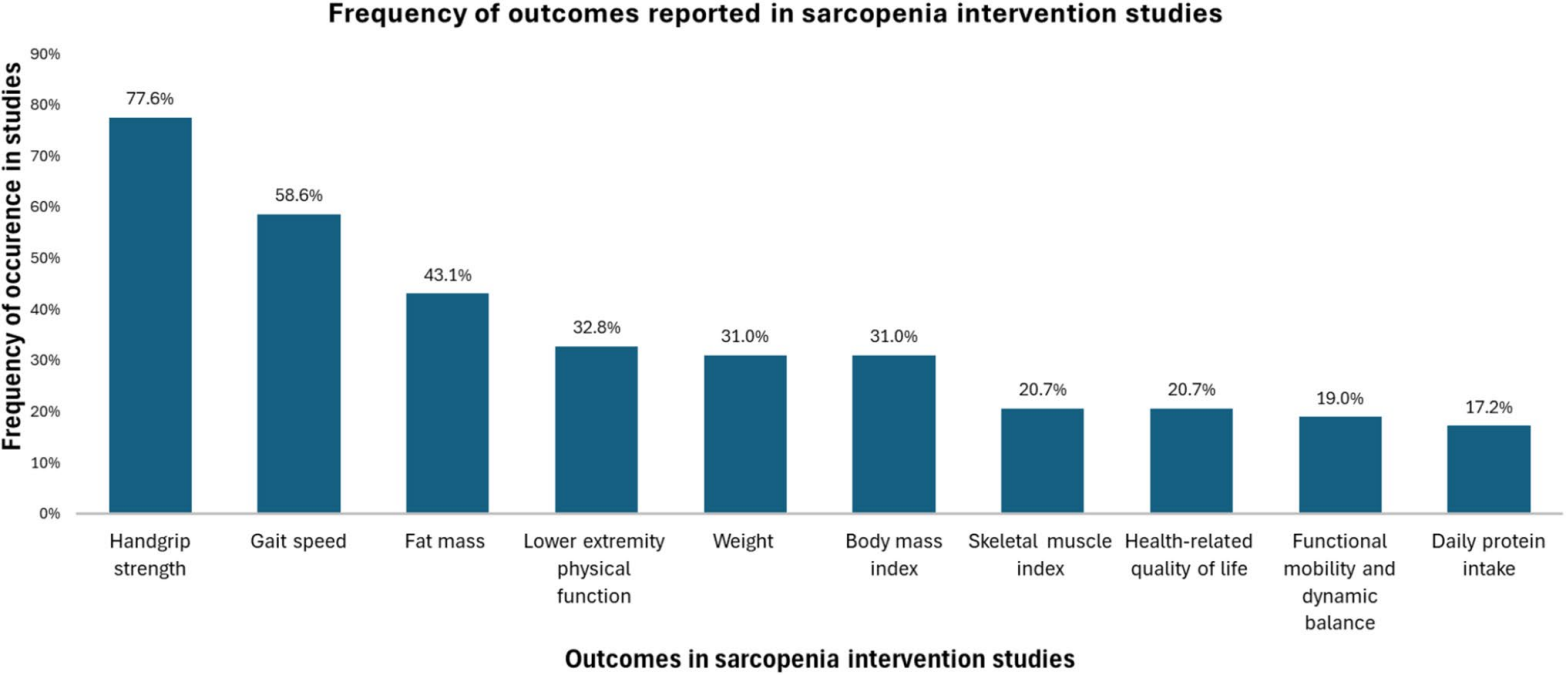
Frequency of reported outcome in sarcopenia intervention studies

Figure 3 shows the most frequently assessed individual outcomes. The most commonly assessed outcome is handgrip strength, reported in 45 studies (77.6%), followed by gait speed (34 studies, 58.6%) and fat mass (25 studies, 43.1%), followed by lower extremity physical function (19 studies, 32.8%), followed by weight and body mass index (BMI) (18 studies, 31.0%). Lesser commonly studied outcomes include skeletal muscle index and health-related quality of life, both assessed in 12 studies (20.7%), functional mobility and dynamic balance, measured in 11 studies (19.0%), and daily protein intake, reported in 10 studies (17.2%).

Table 3 shows all the terms used in the results for the three most frequently reported outcome categories (i.e. muscle mass, muscle strength, and physical performance).

**Table 3:** Overview and frequency of specific variables included in the three most frequently outcomes group

Categories	Name of outcome in studies	Number of studies reporting the individual outcome	Number of studies reporting the individual outcome as a primary outcome
Muscle mass (n = 50)	Skeletal muscle index	12 (20.7%)	1 (1.7%)
	Appendicular skeletal muscle mass index	9 (15.5%)	2 (3.4%)
	Fat-free mass	8 (13.8%)	4 (6.9%)
	Lean body mass	8 (13.8%)	4 (6.9%)
	Skeletal muscle mass	8 (13.8%)	3 (5.2%)
	Appendicular lean body mass	6 (10.3%)	3 (5.2%)
	Appendicular muscle mass	6 (10.3%)	3 (5.2%)
	Calf circumference	6 (10.3%)	0 (0.0%)
	Appendicular skeletal muscle mass	4 (6.9%)	3 (5.2%)
	Muscle mass	4 (6.9%)	0 (0.0%)
	Relative skeletal muscle mass index	4 (6.9%)	3 (5.2%)
	Leg muscle mass	3 (5.2%)	1 (1.7%)
	Muscle quality	3 (5.2%)	0 (0.0%)
	Skeletal muscle density	2 (3.4%)	0 (0.0%)
	Upper limb muscle mass	2 (3.4%)	1 (1.7%)
	Waist circumference	2 (3.4%)	0 (0.0%)
	Fat-free arm	1 (1.7%)	0 (0.0%)
	Fat-free leg	1 (1.7%)	0 (0.0%)
	Fat-free mass index	1 (1.7%)	0 (0.0%)
	Hip circumference	1 (1.7%)	0 (0.0%)
	Lower limb muscle mass	1 (1.7%)	0 (0.0%)
	Lower limb muscle morphology: Pennation angle	1 (1.7%)	1 (1.7%)
	Lower limb muscle morphology: Fascicle length	1 (1.7%)	1 (1.7%)
	Lower limb muscle morphology: Vastus lateralis	1 (1.7%)	1 (1.7%)
	Lower limb muscle morphology: VL cross-sectional area	1 (1.7%)	1 (1.7%)
	Lower limb muscle quality	1 (1.7%)	0 (0.0%)
	Mid-Thigh muscle quality	1 (1.7%)	1 (1.7%)
	Quadriceps cross-selectional area	1 (1.7%)	1 (1.7%)
	Skeletal muscle area	1 (1.7%)	0 (0.0%)
	Skeletal muscle cross-selectional area	1 (1.7%)	1 (1.7%)
	Tested leg muscle mass	1 (1.7%)	0 (0.0%)
	Thigh circumference	1 (1.7%)	0 (0.0%)
	Thigh muscle volume	1 (1.7%)	1 (1.7%)
Muscle strength (n = 50)	Handgrip strength	45 (77.6%)	12 (20.7%)
	Functional lower extremity strength	7 (12.1%)	0 (0.0%)
	Knee extension strength	4 (6.9%)	0 (0.0%)
	Lower limb muscles strength	4 (6.9%)	1 (1.7%)
	Leg strength	2 (3.4%)	1 (1.7%)
	Peak torque for the LEG extension	2 (3.4%)	2 (3.4%)
	Back strength	1 (1.7%)	0 (0.0%)
	Bilateral leg extensors strength	1 (1.7%)	0 (0.0%)
	Maximal isometric muscle strength	1 (1.7%)	0 (0.0%)
	Maximum dynamic muscle strength on the knees	1 (1.7%)	0 (0.0%)
	Maximum isometric contractions	1 (1.7%)	0 (0.0%)
	Maximum single push weight (bilateral leg press)	1 (1.7%)	1 (1.7%)
	Quadriceps strength	1 (1.7%)	0 (0.0%)
Physical performance (n = 46)	Gait speed	34 (58.6%)	7 (12.1%)
	Lower extremity physical function	19 (32.8%)	4 (6.9%)
	Functional mobility and dynamic balance	11 (19.0%)	1 (1.7%)
	Postural mobility/Stability and balance control	5 (8.6%)	0 (0.0%)
	Lower extremity muscle endurance	3 (5.2%)	1 (1.7%)
	Balance and fall risk	2 (3.4%)	0 (0.0%)
	Disability status	1 (1.7%)	0 (0.0%)
	Functional fitness	1 (1.7%)	0 (0.0%)
	Functional status	1 (1.7%)	0 (0.0%)
	Lower extremity function	1 (1.7%)	0 (0.0%)
	Neuromuscular response time	1 (1.7%)	1 (1.7%)
	Postural control/Dynamic stability test	1 (1.7%)	0 (0.0%)
	Postural stabilisation capacity: Sway Path	1 (1.7%)	0 (0.0%)
	Postural stabilisation: Sway area	1 (1.7%)	0 (0.0%)
	Sensory motor control	1 (1.7%)	0 (0.0%)
	Spatial Distance	1 (1.7%)	0 (0.0%)
	Stay Time	1 (1.7%)	0 (0.0%)
	Walking ability	1 (1.7%)	1 (1.7%)

#### Primary outcomes

On average, 2.1 (± 1.9) outcomes were measured in the articles, with a minimum of 1 outcome and a maximum of 8 outcomes. The most frequently assessed primary outcome group was muscle mass changes, reported in 23 studies (39.7%), followed by muscle strength (16 studies, 27.6%) and physical performance (13 studies, 22.4%). Other outcome groups, including physical status, biomarkers, activities of daily living, psychological status, fat mass, quality of life, bone mass, and nutritional outcomes, were less commonly evaluated. Cognitive function was never assessed as a primary outcome (Fig. 2).

Among individual primary outcomes, handgrip strength was the most frequently reported (12 studies, 20.7%), followed by gait speed (7 studies, 12.1%). Other commonly assessed outcomes included fat-free mass, appendicular skeletal muscle mass, and lower extremity physical performance (4 studies, 6.9% each). Additional details on primary outcome groups are provided in Table 3.

Considering the type of intervention proposed, among interventional studies involving exercise (n = 20), the most frequently assessed outcomes were muscle strength (19 studies, 95.0% of all exercise-based studies), physical performance (17 studies, 85.0%), and muscle mass (15 studies, 75.0%). For nutritional interventions (n = 14), the most measured outcomes were muscle mass (11 studies, 78.6% of all nutrition-based studies), biomarkers (11 studies, 78.6%), and muscle strength (10 studies, 71.4%). In studies combining exercise and nutrition (n = 17), both muscle mass and muscle strength were assessed in 100% of cases (17 studies), followed by physical performance in 14 studies (82.4%). Among studies using pharmacological interventions (n = 4), muscle mass and physical performance were evaluated in all studies (4 studies, 100.0%), while muscle strength, fat mass, and physical status were assessed in 3 studies (75.0%) (Table 4).

**Table 4:** Most frequently used outcome groups per intervention

	Exercises n = 20	Nutrition n = 14	Combined n = 17	Pharmacological treatments n = 4	Other n = 3
1	Muscle strength (n = 19;95.0%)	Muscle mass (n = 11;78.6%)	Muscle mass (n = 17;100.0%)	Muscle mass (n = 4;100.0%)	Muscle mass (n = 2;66.7%)
2	Physical performance (n = 17;85.0%)	Biomarkers (n = 11;78.6%)	Muscle strength (n = 17;100.0%)	Physical performance (n = 4;100.0%)	Muscle strength (n = 2;66.7%)
3	Muscle mass (n = 15;75.0%)	Muscle strength (n = 10;71.4%)	Physical performance (n = 14;82.4%)	Muscle strength (n = 3;75.0%)	Physical performance (n = 2;66.7%)
4	Nutritional outcome (n = 9;45.0%)	Physical performance (n = 9;64.3%)	Nutritional outcome (n = 11;64.7%)	Fat mass (n = 3;75.0%)	Fat mass (n = 1;33.3%)
5	Fat mass (n = 9;45.0%)	Nutritional outcome (n = 9;64.3%)	Fat mass (n = 8;47.1%)	Physical status (n = 3;75.0%)	Biomarkers (n = 1;33.3%)
6	Biomarkers (n = 7;35.0%)	Fat mass (n = 7;50.0%)	Other outcome (n = 7;41.2%)	Biomarkers (n = 1;25.0%)	Physical status (n = 1;33.3%)
7	Physical status (n = 5;25.0%)	Quality of life (n = 6;42.9%)	Biomarkers (n = 6;35.3%)	Other outcome (n = 1;25.0%)	Bone mass (n = 1;33.3%)
8	Other outcome (n = 5;25.0%)	Physical status (n = 5;35.7%)	Physical status (n = 6;35.3%)	Quality of life (n = 1;25.0%)	Nutritional outcome (n = 0;0.0%)
9	Activities of daily living (n = 3;15.0%)	Other outcome (n = 5;35.7%)	Quality of life (n = 5;29.4%)	Bone mass (n = 1;25.0%)	Other outcome (n = 0;0.0%)
10	Quality of life (n = 2;10.0%)	Activities of daily living (n = 2;14.3%)	Activities of daily living (n = 5;29.4%)	Nutritional outcome (n = 0;0.0%)	Quality of life (n = 0;0.0%)
11	Cognitive function (n = 2;10.0%)	Psychological status (n = 2;14.3%)	Bone mass (n = 2;11.8%)	Activities of daily living (n = 0;0.0%)	Activities of daily living (n = 0;0.0%)
12	Bone mass (n = 1;5.0%)	Bone mass (n = 1;7.1%)	Psychological status (n = 2;11.8%)	Psychological status (n = 0;0.0%)	Psychological status (n = 0;0.0%)
13	Psychological status (n = 1;5.0%)	Cognitive function (n = 0;0.0%)	Cognitive function (n = 2;11.8%)	Cognitive function (n = 0;0.0%)	Cognitive function (n = 0;0.0%)

Outcomes were also stratified according to geographic location, as detailed in Supplementary Table S5. The top three outcomes across all continents remain muscle mass, muscle strength, and physical performance.

#### Safety outcomes

Regarding safety outcomes, the most frequently reported were adverse events (8 studies, 13.8%), serious adverse events (3 studies, 5.2%), liver function tests (3 studies, 5.2%), and kidney function assessments (3 studies, 5.2%) (supplementary material Tables S6 and S7).

## Discussion

This scoping review is the first to comprehensively identify the outcomes measured in randomized controlled trials (RCTs) on sarcopenia as a first step towards the development of a Core Outcome Set (COS) specific to this disease. It highlights a high heterogeneity in reported outcomes, posing a major challenge for measurement standardization, limiting the feasibility of meta-analyses, and complicating the interpretation and comparison of results across studies. With the increasing number of RCTs on sarcopenia, harmonizing outcomes and measurement tools is a key priority to enhance comparability of results and study reproducibility.

This review brings out that most frequently assessed outcomes in RCTs are muscle mass, muscle strength, and physical performance, which align with the definitions and severity of sarcopenia according to EWGSOP2 definition [1] and to AWGS 2019 definition [28]. Consequently, these criteria are often selected as primary outcomes in studies. However, a concerning finding is the high number of distinct outcomes (n = 253), including 33 different terminologies used to describe muscle mass for example.

This variability also extends to the measurement tools, leading to heterogeneous assessment of interventions and hindering the development of consistent clinical recommendations. For instance, among the three most frequently measured outcomes, handgrip strength was assessed using more than 18 different tools, with some data remaining unspecified. The most commonly used instrument was the Jamar hydraulic hand dynamometer, employed in 37.9% of studies. Gait speed was evaluated using more than five different tools, with the 4-m walking test being the most frequently used (29.3% of studies). In contrast, fat mass was measured using only two methods: bioelectrical impedance analysis BIA (22.4%) and dual-energy X-ray absorptiometry DXA (20.7%). These results demonstrated the importance of standardizing not only outcomes, but also tools used to report those outcomes.

A particularly surprising result was the number of primary outcomes reported per study. Only 58.6% of studies had a single primary outcome, while some reported up to eight. Moreover, most studies did not specify whether their sample size was designed to detect a significant difference for all primary outcomes, raising concerns about statistical power and the reliability of findings.

In addition, it is interesting to note that sarcopenia itself was only assessed as an outcome in four studies (Mori et al. [29], Chiang et al. [30], Chen et al. [31], and Lu et al. [32]), representing 6.9% of the included studies. Most interventions, regardless of type, focused on individual components of sarcopenia, such as muscle mass and strength, rather than assessing whether the intervention reversed the disease itself. The studies mainly considered these measures as continuous variables, without assessing sarcopenia in a binary way (i.e. participants with sarcopenia vs. participants without sarcopenia).

Moreover, it is noteworthy that major clinical outcomes (e.g., fractures, hospitalization, and mortality) are underrepresented in this scoping review. Specifically, only six studies [33–38] assessed the risk of falling, the number of falls, or fall-related injuries. Mortality was reported in only one study [33]. Additionally, key outcomes such as hospitalization risk, length of hospital stay, and fracture incidence [10] were not evaluated in any of the 58 studies included in this review. This gap limits the ability to assess the long-term benefits of interventions beyond the components of sarcopenia, making it difficult to translate research findings into meaningful clinical practice. These critical outcomes should be considered a key area for future prioritization in sarcopenia research due to their high clinical relevance.

The lack of consensus further increases the cost of clinical trials, due to the diversity of evaluated parameters and the absence of a standardized methodological framework [20]. This review highlights the urgent need to develop a COS specific for sarcopenia. COS improves comparability between trials, eliminate redundant criteria, and increase clinical relevance. By standardizing outcome measures, COS facilitate data pooling and enables high-quality meta-analysis using the same outcomes and tools. This strengthens available evidence base without the need for excessive new trials, thereby saving time and resources.

The urgent need for a COS aligns with broader efforts to enhance the methodological rigor of sarcopenia trials. Recognizing these challenges, the European Society for Clinical and Economic Aspects of Osteoporosis and Osteoarthritis (ESCEO) emphasized in 2020 the need to rethink clinical trial design to maximize the chances of successful treatments [39]. The adoption of a robust methodology, including targeted inclusion criteria, standardized assessments, and consideration of patients’ perspectives in outcome evaluation, could facilitate the development of effective treatments to prevent and manage sarcopenia in older adults.

This review has several methodological strengths. It follows a high quality, rigorous approach, adhering to PRISMA guidelines, and includes an exhaustive search across multiple databases. Additionally, it incorporates all interventions targeting sarcopenia and considers various consensual definitions of sarcopenia, ensuring a comprehensive and globally applicable analysis. However, certain limitations should be acknowledged. First, we only included published trials and did not include ongoing trials listed on ClinicalTrials.gov, which may limit the completeness of the results. Second, it is possible that the number of extracted outcomes was overestimated, as some variables may have been measured primarily as confounding factors rather than as true study outcomes. However, to mitigate the risk of misclassification, only variables that were measured both before and after the intervention were included in this scoping review. Third, most of the included studies were conducted in Asia (57.4%) and Europe (24.1%). This geographic imbalance may influence the findings and limits the external validity and global generalizability of this scoping review. Although there appear to be differences in the frequency of outcomes by continent, these variations are likely due to the types of interventions studied rather than the location itself. Furthermore, substantial heterogeneity in study designs, populations, and contexts can be expected within each continent. For example, 65% of exercise-based interventions were conducted in Asia, which may partly explain the observed distribution of results in this region. Finally, we did not assess the methodological quality of the included RCTs. However, the aim of our review was not to analyze the effectiveness of the interventions, where methodological robustness and potential bias would play a central role, but rather to summarize and report the outcomes measured in these studies.

These results will be instrumental for the next steps in the development of the sarcopenia-specific COS [20]. Future efforts will include interviews with patients to incorporate their views. This step is crucial for improving clinical relevance and ensuring that measured outcomes reflect the true impact of interventions on quality of life. After conducting patient interviews, we will perform a Delphi study. First, an initial online survey will be sent to experts and patients. Then, we will revise any items for which there is no consensus and distribute the adapted survey for a second round (two-round Delphi study). Finally, we will convene a working group of experts to discuss the Delphi study results. We will publish and disseminate the finalized COS to promote its widespread adoption and standardization in sarcopenia research and clinical practice.

## Conclusion

In conclusion, this review highlights the significant variability in outcomes and measurement tools used in clinical trials for sarcopenia and underlines the need for a collective effort to harmonize practices. The inconsistent responses of certain outcome measures to different intervention modalities warrant further methodological exploration, which can be better addressed through recent network meta-analysis studies [16, 40, 41]. These studies, focusing on intervention efficacy, are crucial for elucidating the mechanisms behind such contradictions and enhancing the generalizability of findings. The development of a Core Outcome Set (COS) for sarcopenia intervention would improve the comparability of trials, allow for the synthesize of results using meta-analysis, optimize the use of clinical research resources and accelerate the integration of new treatments for sarcopenia. The adoption of standardized and validated outcomes is a priority to ensure that studies are more robust, reproducible, and clinically relevant. However, the implementation of the COS into clinical practice requires a strategic approach, including stakeholder engagement, interoperability with existing protocols, and phased implementation. While the COS is not mandatory, its development represents a significant step toward improving research practices. We hope that the COS will be incorporated into future guidelines for the management of sarcopenia and adopted by regulatory authorities to standardize clinical trials. Ultimately, the COS aims to improve clinical practice, facilitate the integration of new treatments, and enhance the overall quality of care for individuals affected by sarcopenia.

## Supplementary Information

Below is the link to the electronic supplementary material.

Supplementary file1 (DOCX 80 KB)

Supplementary file2 (DOCX 21 KB)

Supplementary file3 (XLSX 99 KB)

Supplementary file4 (DOCX 187 KB)

Supplementary file5 (DOCX 17 KB)

Supplementary file6 (DOCX 32 KB)

Supplementary file7 (XLSX 95 KB)

Supplementary file8 (XLSX 129 KB)

Supplementary file9 (XLSX 420 KB)

## Data Availability

The data supporting this study are included within the article and/or its supporting materials. All data are freely available in Open Access via the Open Science Framework: https://osf.io/f4zks/.
