# Supplementary material for: Towards a core outcome set for sarcopenia intervention studies: a scoping review identifying the most frequently reported outcomes across randomized controlled trials in sarcopenia
Source: Eur Geriatr Med. 2025 Aug 12;16(6):2033–45. doi: 10.1007/s41999-025-01285-x (PMC12743691; doi:10.1007/s41999-025-01285-x)
Supplement: Supplementary file 5 — Supplementary file5 (DOCX 17 KB) [file 41999_2025_1285_MOESM5_ESM.docx]

**Table S5.** Most frequently used outcome groups per continent.

|  | Asia n=31 | Europe n=17 | America n=7 | Other n=3 |
| --- | --- | --- | --- | --- |
| 1 | Muscle strength (n=28;90.3%) | Muscle mass (n=14;82.4%) | Muscle mass (n=7;85.7%) | Muscle mass (n=3;100.0%) |
| 2 | Muscle mass (n=26;83.9%) | Muscle strength (n=14;82.4%) | Physical performance (n=6;85.7%) | Muscle strength (n=3;100.0%) |
| 3 | Physical performance (n=23;74.2%) | Physical performance (n=14;82.4%) | Muscle strength (n=5;71.4%) | Physical performance (n=3;100.0%) |
| 4 | Nutritional outcomes (n=18;58.1%) | Biomarkers (n=11;64.7%) | Fat mass (n=4;57.1%) | Fat mass (n=3;100.0%) |
| 5 | Fat mass (n=15;48.4%) | Physical status (n=8;47.1%) | Biomarkers (n=3;42.9%) | Other outcomes (n=3;100.0%) |
| 6 | Biomarkers (n=11;35.5%) | Nutritional outcomes (n=7;41.2%) | Physical status (n=3;42.9%) | Nutritional outcomes (n=2;66.7%) |
| 7 | Physical status (n=9;29.0%) | Fat mass (n=6;35.3%) | Nutritional outcomes (n=2;28.6%) | Biomarkers (n=1;33.3%) |
| 8 | Other outcomes (n=9;29.0%) | Other outcomes (n=5;29.4%) | Other outcomes (n=1;14.3%) | Bone mass (n=1;33.3%) |
| 9 | Quality of life (n=7;22.6%) | Quality of life (n=5;29.4%) | Quality of life (n=1;14.3%) | Cognitive function (n=1;33.3%) |
| 10 | Activities of daily living (n=6;19.4%) | Activities of daily living (n=4;23.5%) | Bone mass (n=1;14.3%) | Physical status (n=0;0.0%) |
| 11 | Bone mass (n=2;6.5%) | Bone mass (n=2;11.8%) | Psychological status (n=1;14.3%) | Quality of life (n=0;0.0%) |
| 12 | Psychological status (n=2;6.5%) | Psychological status (n=2;11.8%) | Activities of daily living (n=0;0.0%) | Activities of daily living (n=0;0.0%) |
| 13 | Cognitive function (n=1;3.2%) | Cognitive function (n=2;11.8%) | Cognitive function (n=0;0.0%) | Psychological status (n=0;0.0%) |
