# Supplementary material for: Towards a core outcome set for sarcopenia intervention studies: a scoping review identifying the most frequently reported outcomes across randomized controlled trials in sarcopenia
Source: Eur Geriatr Med. 2025 Aug 12;16(6):2033–45. doi: 10.1007/s41999-025-01285-x (PMC12743691; doi:10.1007/s41999-025-01285-x)
Supplement: Supplementary file 6 — Supplementary file6 (DOCX 32 KB) [file 41999_2025_1285_MOESM6_ESM.docx]

**Table S6.** Outcomes reported in the 58 included studies.

| References | Primary outcomes | Secondary outcomes | Safety outcomes |
| --- | --- | --- | --- |
| Exercise-based intervention (20 studies) | | | |
| Chen et al. Exp Gerontol 2018 | Skeletal muscle mass, Appendicular skeletal muscle mass, Weight, Fat mass, Pulmonary function (Peak expiratory flow, Forced vital capacity), Chronic proinflammatory cytokine concentration (High-sensitivity C-reactive protein (hs-CRP), Interleukin-6 (IL-6), Tumor necrosis factor-alpha (TNF-α)) | Handgrip strength, Back strenght, Visceral fat area, Sarcopenia index |  |
| Courel-Ibáñez et al. J Am Med Dir Assoc 2022. | Lower extremity physical function | Handgrip strength, Gait speed, Lower limb strength, Mobility, balance and locomotor performance |  |
| De Sá Souza et al. Int J Environ Res Public Health 2022. | Sleep parameters (sleep latency, REM sleep latency, total sleep time, sleep efficiency, WASO, apnea, AHI, SPO2) | Appendicular mass index, Handgrip strength, Isokinetic peak Torque PT, Lower extremity physical function, Anabolic hormones and pro- and anti-inflammatory cytokines (Serum measure triglycerides, total cholesterol, cholesterol fractions and glucose, urea, creatinine, albumin,  total testosterone, cortisol, growth hormone (GH), insulin-like growth factor 1 (IGF-1), tumor necrosis factor-alpha  (TNF-α), interleukin-1 receptor antagonist (IL-1ra), interleukin (IL)-6, and IL-10) |  |
| Flor-Rufino et al. Arch Gerontol Geriatr 2023. | Respiratory function: Respiratory Function, Respiratory sarcopenia, Maximum inspiratory pressure (MIP), Maximum expiratory pressure (MEP) | Skeletal muscle mass index, Handgrip strength, Gait speed, Lower extremity physical performance, Quality of life |  |
| Flor-Rufino et al. Maturitas 2023. | Handgrip strength | Skeletal muscle index, Gait speed, Body weight, Muscle mass, Fat mass, Body mass index, Maximum isometric contractions, Maximum dynamic muscle strength on the knee, Functional performance, Imaging biomarkers (water apparent diffusion coefficient (ADC), water interstitial diffusion coefficient (D), tissue hydration as transversal relaxation time (T2), proton density fat fraction (PDFF), fat/muscle/bone volumes, and macroscopic fatty infiltration) |  |
| Hassan et al. Geriatr Nurs 2016. | Number of falls | Skeletal muscle mass index, Lean body mass, Handgrip strength, Gait speed, Lower extremity physical performance, Fat mass, Cognitive well-being, Fall efficacy, Quality of life, Weight, Body mass index, |  |
| He et al. Arch Gerontol Geriatr 2024 | Handgrip strength | Skeletal muscle density, Skeletal muscle area, Relative skeletal muscle mass, Skeletal muscle interstitial fat area, Skeletal muscle interstitial fat density, Muscle fat infiltration |  |
| Huang et al. Front. Neurol 2023. | Neuromuscular responses | Postural control (dynamic stability test by ProKin 254) |  |
| Li et al. Front Med 2022 | Fall efficacy, Fall events | Mobility, balance, walking ability and fall risk |  |
| Liang et al. Sci rep 2020 | Activities of daily living, Number of fallers | Handgrip strength, Lower extremity physical performance, Gait speed, Balance function, Mobility, balance, walking ability and fall risk | Adverse events |
| Moghadam et al. J Nutr Health Aging 2020 | Plasma myogenic markers: Myog rate, Pax3 rate, Pax7 rate and Myf5 | Skeletal muscle mass, Handgrip strength, Fat mass, Cardiorespiratory fitness, Weight, Body mass index, Upper body power, Lower body power |  |
| Piastra et al. Biomed Res Int 2018 | Handgrip strength | Skeletal muscle mass, Skeletal muscle mass index, Sway Path, Sway area, Stay time, Spatial Distance |  |
| Rezaei et al. BMC Sports Sci Med Rehabil 2024. | Serum levels of myostatin (ng/L): Blood test Myostatin, Follistatin, growth differentiation factor 15 (GDF-15), C-terminal agrin fragment (CAF) | Skeletal muscle mass, Appendicular skeletal muscle mass, Calf circumference, Handgrip strength, Lower body power, balance and endurance, Gait speed, Mobility, balance, walking ability and fall risk, Standing balance, Fat mass, Blood test, Body mass index, Weight |  |
| Seo et al, Int J Environ Res Public Health 2021 | Muscle quality and muscle growth factors (i.e., GDF-8, GDF-15, activin A, follistatin) | Appendicular skeletal muscle mass, Fat-free mass, Waist circumference, Hip circumference, Maximal isometric muscle strength, Handgrip strength, Functional fitness, Gait speed, Body fat percentage, Fat mass, Bone mineral density, Weight, Body mass index |  |
| Valdes-Badilla et al. BMC Public Health 2023 | Fat-free mass, Fat mass, Handgrip strength, Leg strength, Mobility, balance, walking ability and fall risks, Gait speed | Body weight |  |
| Wang et al. J Nutr Health Aging 2022 | Activities of daily living | Handgrip strength, Gait speed, Lower extremity physical performance (SPPB), Mobility, balance, walking ability and fall risks |  |
| Wei et al. Int J Environ Res Public Health 2022. | Skeletal muscle mass at the 3rd lumbar vertebra (L3 SMA), skeletal muscle interstitial fat area at the 3rd lumbar vertebra (L3 SMFA), and relative skeletal muscle mass index | Handgrip strength, Gait speed, Skeletal muscle cross-selectional area, Skeletal muscle density, Skeletal muscle interstitial fat area, Skeletal muscle interstitial fat density, Relative muscle mass index, Muscle fat infiltration | Daily records, Blood samples (ESR, haemogram, creatinine, creatine kinase, urea, ALT, AST) |
| Yuenyongchaiwat et al. Clin Gerontol 2023. | Depressive symptoms | Inflammatory profiles (serum levels of IL-6 and TNF-α) |  |
| Yuenyongchaiwat et al. Eur J Phys Rehabil Med2022 | Cardio-respiratory performance, Physical activity | Skeletal muscle mass index, Handgrip strength, Gait speed, Maximal inspiratory pressure, Maximal expiratory pressure, Step count |  |
| Zhu et al. Eur J Ageing 2019. | Handgrip strength | Physical performance [balance, gait speed, timed-up-and-go test, and five-times-sit-to-stand test], Muscle mass, Handgrip strength, Lower-limb muscle strength, Gait speed, Physical performance (TUGT), Physical performance (FTSST), Physical performance (balance test), Weight |  |
| Nutrition-based intervention (14 studies) | | | |
| Bauer et al. Aging Clin Exp Res 2020. | Kidney function: estimated glomerular filtration rate eGFR |  | Gastrointestinal tolerability, adverse events, gamma-GT, ASAT, ALAT, vitamin D, PTH and calcium levels |
| Bauer et al. J Am Med Dir Assoc 2015. | Handgrip strength | Physical function (chair rise test, gait speed, and balance score), Chair rise test, Gait speed, Balance score, Appendicular muscle mass, Self-reported physical activity, Activities of daily living, Health-related quality of life, Product compliance, Dietery assessment, Serum 25-OH hydroxy-vitamin D, Insulinlike growth factor 1, Body mass index | Safety assessment |
| Bo et al. Clin Nutr 2019 | Appendicular skeletal muscle mass, Relative skeletal muscle mass index, Handgrip strength, Quality of life | Gait speed, Mobility, balance, walking abality and fall risk, Lower extremity function, Fat mass, Blood biomedical indexes, Body weight, Body mass index, Dietary energy intake, Dietary protein intake, Dietary fat intake, Dietary carbonhydrate intake |  |
| Cramer et al. J Am Med Dir Assoc 2016. | Peak torque (PT) for the LEG extension; maximal voluntary isokinetic | Handgrip strength, Gait speed, Fat mass, Leg muscle mass (LMM), Tested leg muscle mass (TLMM), Muscle quality, Daily energy intake, Daily protein intake, Body mass index, Body weight, Compliance, Serum 25-OH vitamin D |  |
| Hill et al. Calcif Tissue Int 2019. | Biochemical measures (Serum osteocalcin OC (μg/L), Serum procollagen type 1 amino-terminal propeptid P1NP (ng/ml), Serum Ca (corrected for albumin)) and Bone mineral density | Serum 25(OH)D, Parathyroid hormone (PTH), Resorption (carboxy-terminal collagen crosslinks; CTX), and Serum IGF-1. |  |
| Liberman et al. Aging Clin Exp Res 2019. | Chronic low-grade inflammatory profile (CLIP): IL-8, IL-1RA, sTNFR1, IL-6 | Appendicular lean mass, Handgrip strength, Lower extremity physical performance, Fat mass, Physical activity, CRP, Pre-albumin, 25-hydroxyvitamin(OH)D, Dietary vitamin D intake, Dietary protein intake |  |
| Lin et al. Clin Nutr 2021. | Appendicular muscle mass index, Handgrip strength, and Gait speed | Fat-free mass, Fat mass, Plasma biochemical parameters (AST, ALT, TC, TG, HDL, AC sugar, Cr, urine protein, LDL), Weight, Body mass index, Daily energy intake, Daily protein intake, Daily fat intake, Daily carbohydrate intake |  |
| Nasimi et al. J Am Med Dir Assoc 2020. | Lean mass and appendicular lean mass, Nutritional status and Physical activity level | Skeletal muscle mass index, Calf circumference, Waist circumference, Handgrip strength, Gait speed, Fat mass, Physical activity, Serum 25-hydroxy vitamin D, IGF-1, Serum insulin levels, Insulin resistance, Fasting blood glucose, lipid profile, serum albumin, blood urea nitrogen, creatinine, and calcium, Serum high-sensitive C-reactive, Serum malondialdehy, Quality of life, Body mass index, Nutritional status, Tissue hydratation |  |
| Rondanelli et al. Geriatrics 2018. | Fat free mass | Handgrip strength, Fat mass, Android fat mass, Gynoid fat mass, Serum albimin levels, Reactive C-protein, Biomarkers (total plasma homocysteine levels, serum albumin, total cholesterol and triglycerides level, High sensitivity CRP, erythrocyte sedimentation rate, creatinine and azotemia, glycemia, and complete blood count) |  |
| Rondanelli et al. Nutrients 2022. | Appendicular lean mass | Handgrip strength, Lower extremity physical function (SPPB), Fat mass, Visceral adipose tissue volume, Physical activity, Level of inflammation, Mood assessment, Walking and risk of fall, Quality of life, Activities of daily living, Weight, Body mass index | Blood pressure |
| Takeuchi et al. Geriatr Gerontol Int 2019 | ADL performance | Calf circumference, Arm circumference, Handgrip strength, Serum albumin levels, Nutritional risk status, Body mass index, Compliance |  |
| Tamura et al. Nutrients 2023 | Skeletal muscle mass index (SMI) | Calf circumference, Handgrip strength, Functional lower extremity, Lower extremity physical performance, Cardiorespiratory function, Physical activity, Depression levels, Quality of life, Body weight, Body mass index, Basal metabolic rate | Adverse events |
| Verlaan et al. Clin Nutr 2012. | Appendicular muscle mass and lower-extremity function | Handgrip strength, Gait speed, Lower extremity physical performance (SPPB), Serum 25(OH) D |  |
| Yoshimura et al. Nutr J 2019. | Motor domain of Functional Independence Measure (FIM-M) | Skeletal muscle mass (SMI), Handgrip strength, Serum albumin level, ADL level, Nutritional status, Daily energy intake, Daily protein intake, Stroke severity score, Lower limb paralysis stage |  |
| Combined (exercises+nutrition)-based intervention (17 studies) | | | |
| AzevedoPinheiro et al. Fisioter Mov 2020. | Depression, Loneliness level | Quality of life |  |
| Bernabei et al. BMJ 2022 | Mobility disability | Appendicular lean mass, Handgrip strength, Lower extremity physical performance, Disability status, Mood, Cognitive function, Number of falls, Injurious falls, Quality of life, Body mass index, Nutritional status, Healthcare services, Mortality rate | Serious adverse events, Unexpected adverse events, Adverse events under the supervision |
| Chang et al. Clin Nutr 2021. | Lean mass, Grip strength and Gait speed, Body mass | Lower extremity muscle, Fat mass, Cardiorespiratory endurance, Bone content |  |
| Chiang et al. Foods 2021 | Appendicular skeletal muscle mass index | Leg muscle mass, Muscle mass, Calf circumference, Handgrip strength, Knee extension strength, Gait speed, Fat mass, Clinical laboratory data (ALT, creatinine, prealbumin, 25-hydroxivitamin D, shCRP, fasting blood sugar, insulin, HbA1c, HOMAR-IR, IGF-1), History of fracture and falls, Number of falls, Body mass index, Weight, Dietary energy intake, Dietary carbonhydrate intake, Dietary protein intake, Dietary fat intake, Change in state of sarcopenia status |  |
| Da Cruz Alves et al. Nutrients 2022. | Cross-sectional area (CSA) of the quadriceps muscle and Lower limb muscle strentght | Skeletal muscle mass index, Lower limb muscle quality, Handgrip strength, Gait speed, Cardiorespiratory endurance, Inflammatory cytokines (IL1, IL6, IL8, IL10 and TNF-alpha), Weight, Body mass index |  |
| Han et al. Orthopaedic surgery 2022 | Body composition (Fat-free mass, Upper-extremity mass, Health lower extremity skeletal muscle mass, Injured lower extremity skeletal muscle mass, Skeletal muscle mass, Skeletal muscle mass index, Fat mass), Handgrip strength | Daily quality of life, Recovery of hip function, Pain in the hip area |  |
| Kim et al. JAGS 2012 | Appendicular skeletal muscle mass, Leg muscle mass, Body mass index | Knee extension strength, Gait speed, Body mass index |  |
| Li et al. Applied physiology, nutrition, and metabolism 2021 | Muscle-related indicators (Appendicular skeletal muscle mass and Relative skeletal mass index) | Handgrip strength, Body fat mass, Waist-hip ratio, Visceral fat area, Weight, Body mass index |  |
| Meza-Valderrama et al. Arch Gerontol Geriatr 2024 | Handgrip strength, Lower extremity physical performance and Gait speed | Fat-free mass index, Fat free mass), Fat mass, Fat mass index, High-sensitive C-reactive protein, Quality of life, Activities of daily living, Body mass index, Nutritional status, Malnutrition diagnosis, Adherence |  |
| Monti et al. J Cachexia Sarcopenia Muscle 2023 | Muscle morphology (Lower limb muscle morphology, Vastus lateralis [fascicle length (Lf)] muscle morphology, Vastus lateralis [pennation angle (PA)] muscle morphology, Vastus lateralis [cross-sectional area (CSA)] muscle morphology), Appendicular lean mass muscle | Handgrip strength, Lower extremity physical lower, Serum NfL concentration, C-terminal agrin fragment serum |  |
| Mori et al. J Nutr Health Aging 2022. | Appendicular skeletal muscle mass index | Handgrip strength, Usual walking speed, Knee extension strength, Remission rate of sarcopenia, Dietary intakes, Step number |  |
| Rondanelli et al. J Cachexia Sarcopenia Muscle 2020. | Gait speed | Appendicular muscle mass, Skeletal muscle mass index, Handgrip strength, Lower body leg strength, Mobility, balance, walking ability (TUG), Lower extremity physical performance (SPPB), Rehabilitation intensity, Total blood count, glucose, transaminases, albumin, creatinine, blood urea nitrogen, serum electrolytes, transferrin, and total cholesterol), C-reactive protein and 25-hydroxyvitamin D (25 (OH)D levels, Cognitive functions, Risk of falls, Quality of life, Activities of daily living, Functional independance, Weight, Nutritional status, Daily energy intake, Daily protein intake | Gastrointestinal side effect |
| Tokuda et al. J Am Nutr Assoc 2023 | Skeletal muscle mass | Handgrip strength, Knee extension strength, Gait speed, Quality of life, Daily energy intake, Daily protein intake |  |
| Wang et al. Nutrients 2022 | Skeletal muscle mass | Handgrip strength, Lower body strength, Gait speed, Stability and balance control, Fat mass, Body mass index, Daily energy intake, Daily protein intake |  |
| Yang et al. J Nutr Health Aging 2023 | Handgrip strength | Skeletal muscle mass, Skeletal muscle index, Solt lean mass, Fat-free mass, Muscle quality, Lower limb strength, Gait speed, Tumor necrosis factor-like weak inducer of apoptosis, Interleukin-18, Fasting bloog glucose, Cholesterol, Triglyceride, LDL-cholesterol, HDL-cholesterol, Body weight | Liver function: ALT, AST, Bilirubin, Direct bilirubin, Indirect bilirubin, Bile acid  Kidney function: Blood urea nitrogen, Uric acid, Creatinine, Glomerular filtration |
| Zdzieblik et al. Br J Nutr 2015. | Fat free mass | Weight, Fat mass, Bone mass, Power (knee extension), Isokinetic quadriceps strength |  |
| Zhu et al. Age Ageing 2019. | Gait speed | Appendicular skeletal muscle mass index, Upper limb muscle mass, Lower limb muscle mass, Handgrip strength, Bilateral leg extensors strength, Lower limb strength, Upper limb fat mass, Lower limb fat mass, Cardiorespiratory fitness, Physical activity, Mental-related quality of life, Physical-related quality of life, iADL impairment, Muscle power in the upper extremities | Renal function: serum urea and creatinine, Control of diabetes, Adverse events, Serious adverse events |
| Pharmaceutical intervention (4 studies) | | | |
| Achison et al. J Cachexia Sarcopenia Muscle 2022 | Lower extremity physical performance (SPPB) | Appendicular muscle mass, Handgrip strength, Leg strength, Lower limb strength, Lower extremity physical performance, Gait speed, Cardiorespiratory function, Insulin resistance, Quality of life, Activities of daily living | Adverse events |
| Papanicolaou et al. J Nutr Health Aging 2013. | Muscle strength (1-repetition maximum by bilateral leg press) | Lower extremity physical function (SPPB), Lean body mass, Appendicular lean body mass, Total fat mass, Bone mineral content, Lower extremity power, Gait speed, AM-Pac | Clinical evaluation, Vital signs, Physical examinations, Laboratory safety assessments, ECGs and Liver function test |
| Rooks et al. J Am Geriatr Soc 2017. | Thigh muscle volume | Total lean body mass (LBM), Handgrip strength, Gait speed, Fat mass, Intermuscular fat tissue of the thigh, Cardiorespiratory fitness | Pharmacokinetic, Adverse events |
| Rooks et al. JAMA Netw Open 2019. | Lower extremity physical function (SPPB) | Lean body mass, Appendicular skeletal mass index, Handgrip strength, Gait speed, Fat body mass, Cardiorespiratory function | Standard laboratory tests, Vital signs, Electrocardiogram, Echocardiography, Reports of adverse events (AEs) and serious AEs. |
| Other intervention (3 studies) | | | |
| Lu et al. Jama Netw open 2019. | Appendicular skeletal muscle index, Lower limb strength, and Gait speed | Sarcopenia score |  |
| Min-Kyun Oh et al. J Gerontol A Biol Sci Med Sci 2020. | Walking ability | Handgrip strength, Physical function and walking ability, Cognitive function, Balance and fall risk, Quality of life, Activities of daily living |  |
| Soares Mendes Damasceno et al. J Aging Res 2019. | Muscle strength | Lean body mass, Functional capacity, Body fat percentage, Bone mineral content, Inflammatory mediators |  |
